# MRN- and 9-1-1-Independent Activation of the ATR-Chk1 Pathway during the Induction of the Virulence Program in the Phytopathogen *Ustilago maydis*

**DOI:** 10.1371/journal.pone.0137192

**Published:** 2015-09-14

**Authors:** María Tenorio-Gómez, Carmen de Sena-Tomás, Jose Pérez-Martín

**Affiliations:** Instituto de Biología Funcional y Genómica (CSIC), Salamanca, Spain; Università degli Studi di Milano, ITALY

## Abstract

DNA damage response (DDR) leads to DNA repair, and depending on the extent of the damage, to further events, including cell death. Evidence suggests that cell differentiation may also be a consequence of the DDR. During the formation of the infective hypha in the phytopathogenic fungus *Ustilago maydis*, two DDR kinases, Atr1 and Chk1, are required to induce a G2 cell cycle arrest, which in turn is essential to display the virulence program. However, the triggering factor of DDR in this process has remained elusive. In this report we provide data suggesting that no DNA damage is associated with the activation of the DDR during the formation of the infective filament in *U*. *maydis*. We have analyzed bulk DNA replication during the formation of the infective filament, and we found no signs of impaired DNA replication. Furthermore, using RPA-GFP fusion as a surrogate marker of the presence of DNA damage, we were unable to detect any sign of DNA damage at the cellular level. In addition, neither MRN nor 9-1-1 complexes, both instrumental to transmit the DNA damage signal, are required for the induction of the above mentioned cell cycle arrest, as well as for virulence. In contrast, we have found that the claspin-like protein Mrc1, which in other systems serves as scaffold for Atr1 and Chk1, was required for both processes. We discuss possible alternative ways to trigger the DDR, independent of DNA damage, in *U*. *maydis* during virulence program activation.

## Introduction

When faced with DNA damage, eukaryotic cells activate DNA damage response (DDR) pathways that help to preserve genome integrity and cell viability. Depending on the manner, extent, and cellular context of the DNA damage, the outcomes of DDR signaling range from transient cell cycle arrest coupled with DNA repair to apoptosis or senescence. In parallel, an increasing number of reports show the involvement of DDR programs in cell differentiation, aside from their more conservative role of protecting genome integrity [1]. The most obvious example is the vertebrate adaptive immune system, which requires the programmed induction and subsequent repair of double strand breaks (DSB) during antigen receptor gene rearrangements [2]. A second well-known example, in fungi, is the switching of mating-type in budding and fission yeast, which involves a programmed DSB followed by DNA repair [3, 4]. In other occasions, the induction of DDR in response to non-programmed DNA damage can be used to activate alternative differentiation processes, such as neurite outgrowth in neuronal stem cells [5], or as the transition between yeast and hyphal growth in *Schizosaccharomyces japonicus* [6]. Perhaps the most extreme case supporting a role of DDR in developmental programs is the use of elements from the DDR cascade to control cell cycle regulation during a differentiation process in the absence of any observed DNA damage. In the embryos from the nematode *Caenorhabditis elegans*, differential cell cycle duration starts at the two-cell stage, when the larger anterior blastomere AB divides before the smaller posterior blastomere P1. Interestingly, the delay in P1 cells relies on two conserved elements from DDR cascade, Atl-1 (ATR) and Chk-1 (Chk1), and apparently there is no DNA damage associated with the activation of the DDR cascade during this process [7]. In the same way, during the differentiation of mammalian trophoblast stem (TS) cells, Chk1 prevents cell cycle exit and thereby premature differentiation of TS cells, in the absence of induced DNA damage [8].

Owing to the intricate connections between cell cycle regulation and differentiation processes [9], as well as to the ability of DDR cascade to regulate the cell cycle, it is tempting to hypothesize that DDR cascade may be recruited to modulate cell cycle during developmental processes, even if no DNA damage signal exists; most likely, this would occur through alternative ways of DDR activation. In the present work we aimed to test this hypothesis by studying it during the virulence program of the phytopathogenic fungus *Ustilago maydis*.

The activation of the virulence program in the corn smut fungus *U*. *maydis* involves the mating of a pair of compatible haploid budding cells, which results in an infectious dikaryotic hypha that grows on the plant surface until finding a suitable place to penetrate the plant tissue [10]. A peculiar characteristic of the *U*. *maydis* dikaryotic filament is the sustained G2 cell cycle arrest while growing on the plant surface [11, 12]. This cell cycle arrest is a requisite for the virulence in *U*. *maydis*. Mutant strains unable to arrest the cell cycle cannot effectively infect plants, because their ability to differentiate specific infection structures–the *appressoria—*is severely impaired [13]. As described in other systems, the response to DNA damage in *U*. *maydis* is mediated by the DDR kinase Chk1, activated by the upstream kinase Atr1 [14]. Previous research from our group showed that Chk1 was necessary for the cell cycle arrest establishment observed during the formation of the infective filament [15]. Moreover, during this process, Chk1 is activated via phosphorylation by Atr1 at the same residues which need to be phosphorylated in response to DNA damage [16]. Our work strongly suggests that the differentiation process during the virulence program in *U*. *maydis* involves the Atr1-Chk1 axis. The formation of the infectious hypha in *U*. *maydis and* the induced cell cycle arrest are triggered by the expression of a transcriptional master regulator called b-factor [17]. How this transcription factor can induce the activation of the Atr1-Chk1 cascade in order to arrest the cell cycle is unknown. Here we report our attempts to determine whether DNA damage is associated with the induction of the virulence program in *U*. *maydis*, as well as to define additional elements of the Atr1-Chk1 cascade involved in the control of the cell cycle arrest occurring in the infective filament.

## Materials and Methods

### Strains and growth conditions

*U*. *maydis* strains were derived from FB1 and FB2 genetic backgrounds [18] and are listed in Table 1. Cells were grown in rich medium (YPD), complete medium (CMD) or minimal medium (MMD) [19]. FACS analyses were described previously [20].

**Table 1:** *U*. *maydis* strains used in this study.

Strain	Relevant Genotype	Reference
**FB1**	*a1 b1*	[19]
**FB2**	*a2 b2*	[19]
**AB33**	*a2 Pnar1*:*bW2 Pnar1*:*bE1*	[23]
**AB34**	*a2 Pnar1*:*bW2 Pnar1*:*bE2*	[23]
**UMC19**	*a2 Pnar1*:*bW2 Pnar1*:*bE1 cbx1*::*Pnar*:*cdk1* ^*AF*^ *-myc-cbx*	[16]
**UMC20**	*a2 Pnar1*:*bW2 Pnar1*:*bE1 cbx1*::*Pnar*:*cdk1-myc-cbx*	[16]
**UMT007**	*a1b1 rfa1-GFP*	This study
**UMT011**	*a2 Pnar1*:*bW2 Pnar1*:*bE1 rfa1-GFP*	This study
**UMT012**	*a2 Pnar1*:*bW2 Pnar1*:*bE2 rfa1-GFP*	This study
**UMP210**	*a1b1 Δrec1*	[39]
**UMP211**	*a2b2 Δrec1*	This study
**UMP219**	*a1b1 Δmre11*	[39]
**UMP220**	*a2b2 Δmre11*	This study
**UMT010**	*a2b2 mrc1* ^*1-914*^ *-HA*	This study
**UMT009**	*a2b2 mrc1* ^*1-914*^ *-HA*	This study
**UMP111**	*a1b1 chk1-3GFP*	[15]
**UMT002**	*a1b1 chk1-3GFP Δrec1*	This study
**UMT019**	*a1b1 chk1-3GFP Δmre11*	This study
**UMT014**	*a1b1 chk1-3GFP mrc1* ^*1-914*^	This study
**UMP121**	*a2 Pnar1*:*bW2 Pnar1*:*bE1 Pdik6*:*NLS-GFP*	[16]
**UMT005**	*a2 Pnar1*:*bW2 Pnar1*:*bE1 Pdik6*:*NLS-GFP Δrec1*	This study
**UMT015**	*a2 Pnar1*:*bW2 Pnar1*:*bE1 Pdik6*:*NLS-GFP Δmre11*	This study
**UMT016**	*a2 Pnar1*:*bW2 Pnar1*:*bE1 Pdik6*:*NLS-GFP mrc1* ^*1-914*^	This study

### Plasmid and strain constructions

Plasmid pGEM-T easy (Promega) was used for cloning, subcloning and sequencing of fragments generated by PCR. The oligonucleotides are described in Table 2. To construct the different strains, transformation of *U*. *maydis* protoplasts with the indicated constructions was performed following published procedures [21].

**Table 2:** Oligonucleotide primers used in this study.

Name	Sequence 5’-3’
**RT-PCR**	
**um06368-1**	CATCACTGAGGCTGTGGAAA
**um06368-2**	TTCCAACGAAATGTTGGTCA
**um04529-1**	GCTCCAAGCTCAAAGGTCAC
**um04529-2**	AGGGACGGTATGCATCAAAG
**um03501-1**	CTTGGTACCGTGGCTTCAAT
**um03501-2**	CACGATACGTTCTTCGAGCA
**um01008-1**	TCATCTTTTCGCTGTGCAAC
**um01008-2**	AGGAGGTGGCCTTTGTAGGT
**um11750-1**	GGCAACCCTTTCATCCTGTA
**um11750-2**	TTGGTCACTGGGTCAATGAA
**um06402-1**	TTCACGAAGTGATGGAGCAG
**um06402-2**	CGCAGGGAGGTTGATATTGT
***rec1* deletion allele**	
**Rec1-2**	GCTTAATTAAGCTGGAACTCCACTCTGCTCTAGCTC
**Rec1-3**	GGTGGCCATCTAGGCCGGCATGCTGACGGTGGCGTCAACTGG
**Rec1-4**	ATAGGCCTGAGTGGCCTTGCGCAATCGCCGCTGAAGTTGATC
**Rec1-5**	GGTTAATTAATCGAGTTGGCCTTCTTGTCTGCTGCA
***mre11* deletion allele**	
**Mre11-2**	GCTTAATTAATATTTGCCTGTTGTCTGTGCGTTGAGAACG
**Mre11-3**	GGTGGCCATCTAGGCCTCGCTTGCTCGCACGAAATCAAACTAGATA
**Mre11-4**	GGTGGCCTGAGTGGCCGATTCAGCGAGTCGGCCAAGATGGTGGAGA
**Mre11-5**	GCTTAATTAAAATATCCAGCTGGCTTCGACATTCGACCAA
***mrc1*** ^***1–914***^ **allele**	
**Mrc1-11**	GCTTAATTAACAAGACAGCAGGAGCGCAGACTAGGCCTTG
**Mrc1-12**	GGTGGCCGCGTTGGCCTCCGTCTCTTTGCCTTTGTTCAGGCTTGTC
**Mrc1-13**	GGTGGCCTGAGTGGCCGACGAGGACGAGGACGAGGACGAGGACGAC
**Mrc1-14**	GCTTAATTAAGTCCTGCTGCGTCTCCTGGAAGAAAGCGCC
***rfa1-3GFP* allele**	
**Rfa1-2**	GCTTAATTAAGACGTTCCCGAGGTCAAGTACGAGTTTGTG
**Rfa1-3**	GGTGGCCGCGTTGGCCATATAGGCTCTGATCGCATCCACCAACTCC
**Rfa1-4**	GGTGGCCTGAGTGGCCAGCGGGCGCATGGTTCACATCATAGTTCGC
**Rfa1-5**	GCTTAATTAGGAGCGCGAATTCGGAAAATGCGTGGTTGG

Deletion of *mre11*, *rec1* and *mrc1* genes was done by gene replacement [22]. Briefly, a pair of DNA fragments flanking the corresponding ORF were amplified and ligated to antibiotic resistance cassettes via *Sfi*I sites. The 5’ and 3’ fragments were amplified using the oligonucleotide pairs respectively (Table 2). Each fragment was about 1 kbp in length. Integration of the disruption cassette into the corresponding loci was verified in each case by diagnostic PCR and subsequent Southern blot analysis.

For C-terminal fusion of proteins to fluorescent markers, the adaptation of the *Sfi*I-dependent gene replacement strategy for C-terminal tag [23] was used. To produce Rfa1–3GFP, 5′ and 3′ fragments were digested with *Sfi*I and ligated to a cassette carrying a triple GFP-encoding gene. Chk1-GFP fusion was already described [14].

### RNA analysis

Total RNA was extracted with acidic phenol solution. After extraction, the RNA was cleaned using the High Pure RNA Isolation Kit (Roche Diagnostics GmbH). For qRT- PCR, cDNA was synthesised using the High Capacity cDNA Reverse Transcription Kit (Applied Biosystems) employing 1 μg total RNA per sample. qRT-PCR was performed using the SsoAdvanced Universal SYBR Green Supermix (BioRad) in a CFX96 Real-Time PCR system (BioRad). Reaction conditions were as follows: 3 min 95°C followed by 40 cycles of 10 sec 95°C/10 sec 60°C/30 sec 72°C.

### Plant infections

Pathogenic development of wild type and mutant strains was assayed by plant infections of the maize (Zea mays) variety Early Golden Bantam (Olds seeds) as described before [24].

### Microscopy

Images were obtained using a Nikon Eclipse 90i fluorescence microscope with a Hamamatsu Orca-ER camera driven by Metamorph (Universal Imaging, Downingtown, PA). Images were further processed with Adobe Photoshop CS software.

## Results

### ATR-Chk1 activation is not triggered by impaired bulk DNA replication

**Fig 1:**
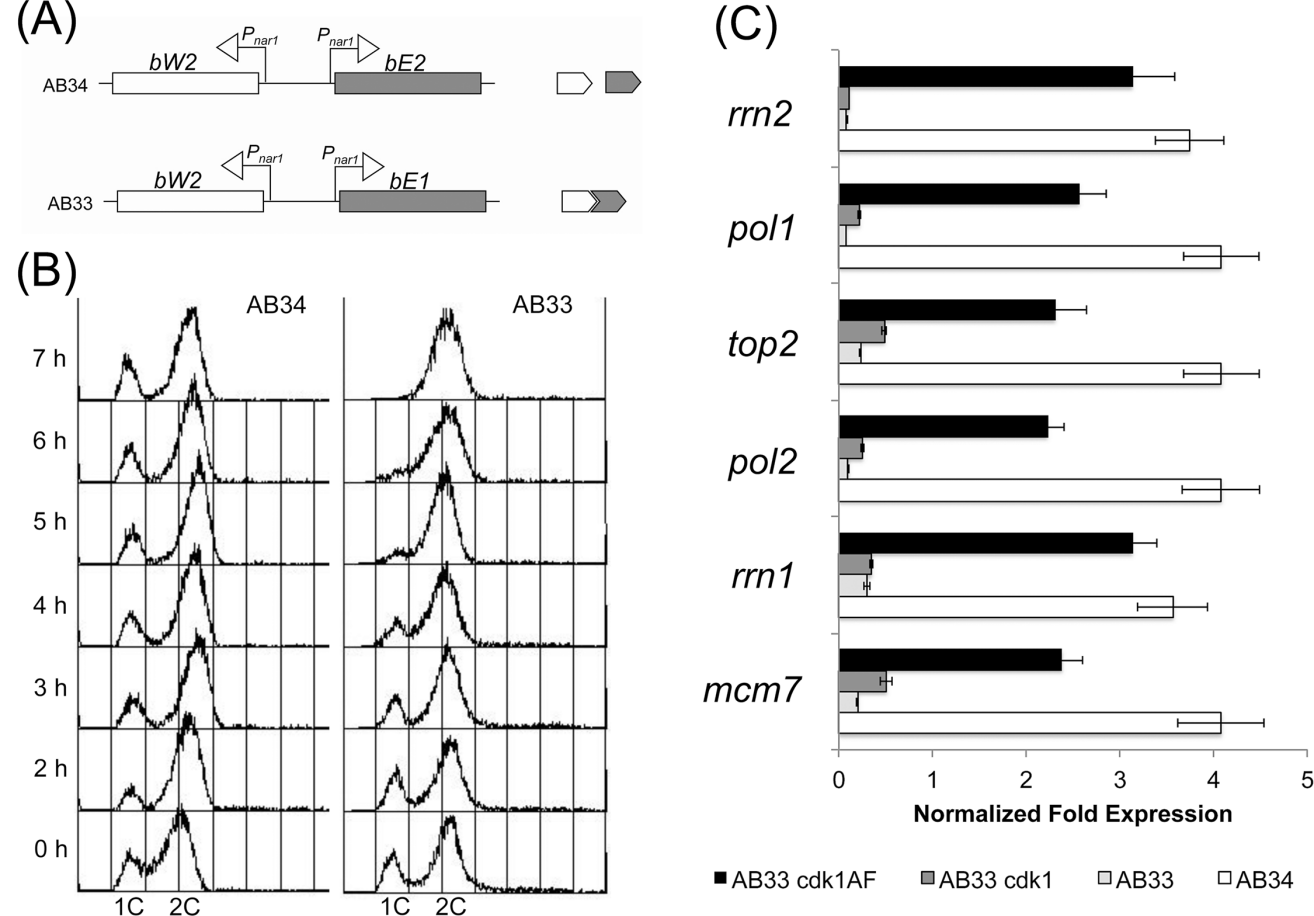
Absence of global replication delay upon activation of the genetic program that leads the formation of the infective filament in *U*. *maydis*. (A) Scheme of the cassettes expressing compatible (AB33) or non-compatible (AB34) b-factor genes. Only the compatible pair is able to form the heterodimer. (B) FACS (Fluorescence-activated cell sorting) analysis of the DNA content of AB34 and AB33 strains growing in inducing (MM-NO_3_) conditions. The period of incubation in testing medium is indicated (hours). (C) Quantitative real time-PCR for the indicated genes in the different strains. RNA was isolated after 6 hours of induction of *nar1* promoter. As internal control the expression of *tub1* (encoding Tubulin α) was used. Each column represents the mean value of four independent biological replicates. Error bars represent the SD; **p<0.01 based on a two-tailed Student´s *t*-test compared to control sample (AB34).

The formation of the infectious dikaryotic hypha in *U*. *maydis* depends on an intricate transcriptional program that primarily involves a transcriptional regulator called b-factor [17]. The production of this master regulator is linked to the mating process that, after cell fusion, leads to the interaction of the two subunits composing the b-factor (bW and bE), each subunit provided by each mating partner. In the laboratory it is possible to bypass the requirement for a mating process for production of the infective filament by using the specialized strain AB33 and its control strain AB34. These strains harbor the compatible *bE1* and *bW2* and non-compatible *bE2* and *bW2* genes under the control of the nitrate-inducible *nar1* promoter, respectively [25] (Fig 1A). Induction of *bE1/bW2* in the AB33 strain growing in medium with nitrate results in the formation of monokaryotic infective filaments that resemble the infectious hypha formed after fusion of compatible haploid cells, including the cell cycle arrest in G2 phase [15].

Previous studies have analyzed the *U*. *maydis* transcriptome in AB33 cells in conditions of expression of b-factor (i.e. forming the infective filament) and found that mRNA levels of several genes involved in DNA replication decrease upon *b*-expression [26]. Among these genes were those encoding components of the replication machinery such as um04529 (*pol1*, DNA-directed DNA polymerase α); um03501 (*top2*, DNA topoisomerase II); um01008 (*pol2*, a subunit of the DNA polymerase ε); and um06402 (*mcm7*). In addition, the expression of two of the components of the ribonucleotide reductase (um06368 and um11750, encoding for the small and large subunits, *rrn2* and *rrn1* respectively), which is required for the synthesis of dNTPs, appears severely down-regulated. The decrease in the levels of any of these proteins would affect the ability of the cells to replicate their DNA, and it could be a source of signals to activate DDR in *U*. *maydis* during the formation of the infective filament. Therefore we decided to address whether cells accumulate in the S-phase at some point during the induction of the infective filament. For that we monitored the DNA content using FACS analysis of cultures of AB33 and AB34 strains for each hour after the induction of the expression of b-factor (Fig 1B). No accumulation of S-phase cells was detectable at these times just before the arrest, suggesting that DNA replication was not compromised during the formation of the b-dependent filament.

Strikingly, our conclusion seems to be contradictory with the published observation of a down-regulation of genes involved in DNA replication upon *b*-expression [27]. However, down-regulation of these genes could be merely a consequence of the b-induced cell cycle arrest. Therefore, we analyzed the expression of the down-regulated genes in conditions of *b*-expression and non-arrested cell cycle. For that we used a *U*. *maydis* strain simultaneously expressing the genes encoding the b-factor as well as an ectopic Cdk1 allele refractory to inhibitory phosphorylation at Tyr15 (*cdk1* ^*AF*^), the ultimate cause of the b-induced G2 cell cycle arrest [15, 28]. In this strain, the *cdk1* ^*AF*^ allele (and a control wild-type *cdk1* allele) was expressed under the *nar1* promoter. As a consequence, in spite of the activation of the b-dependent transcriptional program, the cell cycle was not arrested [13, 15]. We have found, in agreement with a previous report [27], that for all the analyzed genes, the levels of mRNA dramatically decreased upon *b*-expression. However, this decrease seems to be a consequence of the cell cycle arrest: interference with the *b*-induced cell cycle arrest upon expression of the *cdk1* ^*AF*^ allele prevented the decrease in the mRNA levels in all cases (Fig 1C).

### ATR-Chk1 activation seems not to be due to massive DNA damage

**Fig 2:**
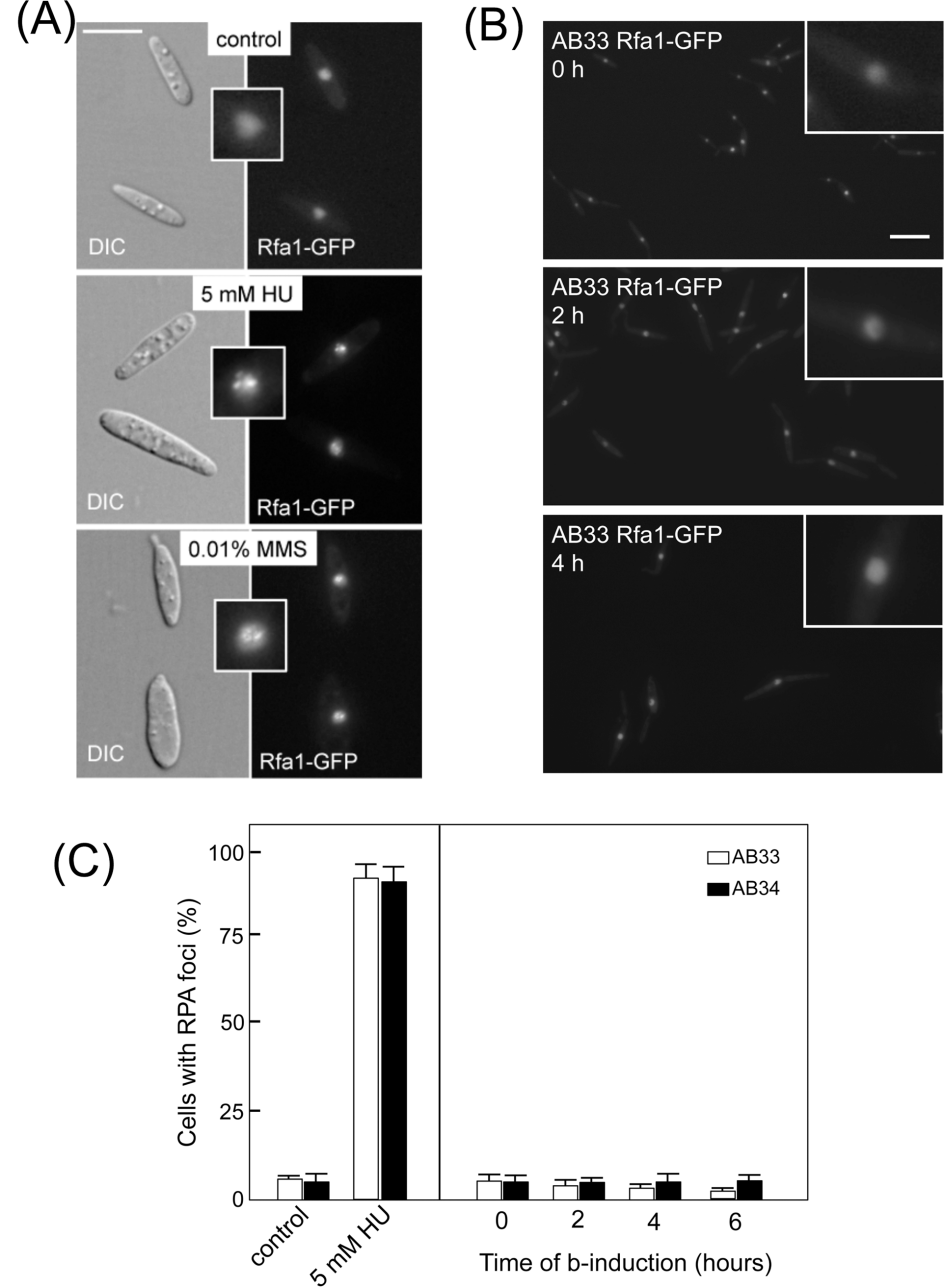
Analysis of RPA foci in the b-induced filament of *U*. *maydis*. (A) Rfa1-GFP foci observed in a strain carrying the endogenous *rfa1* allele tagged with a triple GFP cassette and treated with the indicated genotoxic agents. Insets showed magnification of representative nucleus in each case. Bar: 15 μm (B). Absence of Rfa1-GFP foci after b-induction in the UMT011 strain (AB33 derivative carrying the *rfa1-3GFP* allele). Representative images of cultures at the indicated times are shown. Insets show magnification of representative nucleus in each case. Bar: 10 μm. (C) Quantification of cells showing RPA-GFP foci. The graph shows the result from two independent experiments, counting more than 50 cells each. Means and SDs are shown.

Having no evidence supporting the idea of a global replication collapse as responsible for activating the Atr1-Chk1 axis during the induction of the *b*-dependent filament, we searched for any signal of DNA damage during this process. In a previous report [15], we had tried to use the presence of Rad51-GFP foci as a surrogate marker for the presence of DNA damage [29]. Using this approach we found no evidence of DNA damage during the induction of the *b*-dependent filament. However, since Rad51 acts in only a subset of responses to DNA damage, we sought to use an alternative way to detect any DNA damage signal. The appropriate DNA substrates for checkpoint initiation can be generated by several pathways, but all of them have in common the production of various types of single-stranded DNA (ssDNA) regions that are bound by the single-strand binding protein RPA [30]. RPA-coated ssDNA is instrumental in the recruitment of checkpoint complexes, and can be detected as foci using either immunofluorescence or fluorophore-coupled alleles of components of this protein complex [31]. For this purpose, we constructed a GFP-tagged allele of *rfa1*, encoding one of the subunits from the RPA complex in *U*. *maydis* [32]. A wild-type strain carrying the endogenous *rfa1* gene tagged with GFP showed the presence of nuclear foci in response to DNA damage agents, such as hydroxyurea (HU) and methyl methanesulfonate (MMS) treatment (Fig 2A). We introduced the *rfa1-GFP* allele into the AB33 strain and checked for the presence of nuclear foci during the induction of *b*-dependent filaments. The activation of Chk1 during the formation of the infective filament is transient, with its maximum achieved within 4 hours from b-factor induction [15]. Therefore, we analyzed the presence of RPA foci during this period. Strikingly, we did not observe a higher frequency of RPA foci in cell nuclei with respect to AB34 control strain (Fig 2B and 2C). These results claim against the presence of massive DNA damage during the formation of the *b*-dependent filament as the trigger that activates the Atr1-Chk1 pathway in *U*. *maydis*.

### Characterization of elements upstream of Atr1-Chk1 axis in *U*. *maydis*

The absence of RPA foci does not necessarily preclude the presence of some specific damage in the DNA during the induction of the *b*-dependent filament. It is plausible that in response to *b*-induction, some limited DNA damage could be enough to induce the Atr1-Chk1 cascade, even when this is not detected by the presence of RPA foci. Therefore, we reasoned that the characterization of elements acting upstream of the Atr1-Chk1 cascade would help to define which kind of DNA damage signal, if any, could be involved in the *b*-dependent activation of Chk1 in *U*. *maydis*.

Elements upstream of the Atr1-Chk1 cascade have extensively been investigated in other organisms. While ATR recruitment to the damaged DNA seems to rely just on the presence of RPA-coated ssDNA, its activation needs action from other proteins. This is the case of the PCNA-like complex 9-1-1 (Rad9-Rad1-Hus1), which acts at two distinct levels depending on the species and the cell cycle phase [33]: directly activating ATR by DNA-bound 9-1-1; or indirectly, by recruiting the TopBP1/Dpb11 protein to the damaged sites, which in turn directly activates ATR [30]. Nevertheless not only 9-1-1 is able to assemble TopBP1 to these areas, and there are cases in which this function has also been found in the MRN complex (Mre11-Rad50-Nbs1)[34]. In addition to these complexes, adaptor proteins working as scaffolds are required for the appropriate transmission of the DNA damage signal. For instance, in budding yeast the adaptor proteins Rad9 and Mrc1 are required for checkpoint kinases activation in response to different kind of damages [35–37].

Components from 9-1-1 complex, like Rec1 (the Rad1 ortholog), and from MRN complex, like Mre11, have been previously described for *U*. *maydis* [38, 39]. We queried the NCBI and Broad Institute databases for *U*. *maydis* homologues of TopBP1/Dpb11, *S*. *cerevisiae* Rad9 (*S*. *pombe* Crb2) and Mrc1. With the exception of Rad9/Crb2, we were able to identify in the genome of *U*. *maydis* the putative homologues of TopBP1/Dpb11 (um00290, renamed as Dpb11) and Mrc1 (um06299, renamed as Mrc1).

Neither *rec1* nor *mre11* are essential genes in *U*. *maydis*, and therefore it was possible to construct the respective loss-of-function mutants (See below). In contrast, both Dpb11 and Mrc1 turned out to be essential proteins in *U*. *maydis*, and we were unable to disrupt their respective genes in haploid cells. In other organisms, both Dpb11/TopBP1 and Mrc1/Claspin maintain additional functions during DNA replication [40, 41] that could explain their essential role. In *S*. *cerevisiae*, Dpb11 is also essential but it is possible to separate the critical replication function from the checkpoint activation function by using specific mutants. One of these mutants is *dpb1-11*, which carries a truncated C-terminus immediately after the fourth BRCT domain (W583STOP) [42]. We tried to recreate this mutant in haploid cells introducing a stop codon at the equivalent residue in *U*. *maydis dpb11* (Q846STOP, S1 Fig) with no success, suggesting that this kind of mutation does not recapitulate the same phenotype in *U*. *maydis*. In the case of Mrc1, separation-of-function mutants have also been described for *S*. *cerevisiae MRC1*, consisting in site-specific mutations in all SQ/TQ residues [43]. *U*. *maydis* Mrc1 carries 32 SQ/TQ residues scattered along the entire protein. However, we have found that, in *U*. *maydis*, the *mrc1* ^*1-914*^ allele (S2 Fig), carrying a C-terminal end truncation of Mrc1 resulted into viable cells that do not respond to checkpoint activation (see below).

**Fig 3:**
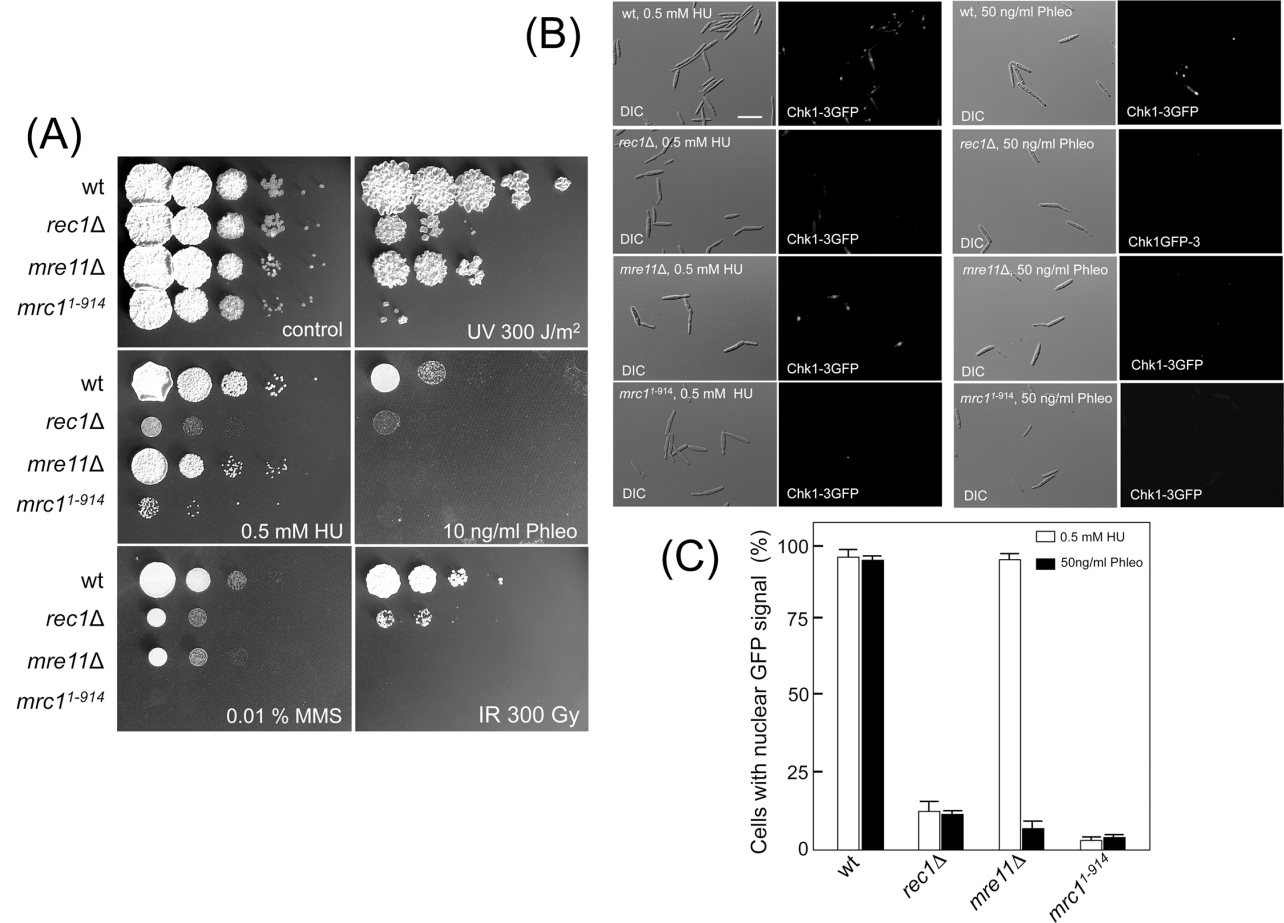
DNA damage response is dependent of Rec1, Mre11 and Mrc1 proteins. (A) Sensitivity of the *rec1*Δ, *mre11*Δ and *mrc1* ^*1-914*^ cells, subject to different types of genotoxic stress (HU, hydroxyurea; MMS, methyl methanesulfonate; Phleo, phleomycin; IR, ionizing radiation). 10-fold dilutions were plated onto YPD medium containing the indicated drug or irradiated with UV light or IR after being plated onto YPD medium. Control plate was incubated for 2 days while treated plates were incubated for 3 days. (B) Nuclear localization of Chk1-GFP after the induction of genotoxic stress (hydroxyurea, HU, and, Phleomycin, Phleo) in the *rec1*Δ, *mre11*Δ and *mrc1* ^*1-914*^ mutant strains. Bar: 15 μm. (C) Quantification of cells showing nuclear GFP signal. The graph shows the result from two independent experiments, counting more than 50 cells each. Means and SDs are shown.

To establish whether 9-1-1, MRN, and Mrc1 are required in *U*. *maydis* for Chk1 activation, first we confronted the respective mutants to different genotoxic insults: UV irradiation, which induces pyrimidine dimers in DNA; HU, which inhibits ribonucleotide reductase and, therefore, affects replication by depletion of deoxynucleotide triphosphates, causing replication fork stalling and collapse; MMS, which induces DNA alkylation; phleomycin, a radiomimetic drug that causes DSB in DNA; and ionizing radiation (IR), which also generates DSB. We have found that all the mutants were sensitive to MMS, to phleomycin and to both UV and gamma irradiation, but only *rec1*Δ and *mrc1* ^*1-914*^ mutants were sensitive to HU (Fig 3A). These results were consistent with different complexes devoted to signaling different forms of DNA damage—MRN, which senses DSBs, and 9-1-1, which senses ssDNA that is produced as a consequence of DNA replication stress or resection [44].

Activation of the DDR in *U*. *maydis* is marked by phosphorylation of Chk1 and by its relocalization into the nucleus [14]. We examined the subcellular localization of GFP-tagged Chk1 in the presence of sub-lethal concentrations of either HU (producing mainly the presence of unreplicated forks) or phleomycin (inducing DSBs) (Fig 3B and 3C). While control cells showed a clear nuclear accumulation of the fluorescent signal in the presence of these DNA damaging agents, the different mutant strains failed to accumulate the fluorescent signal into the nucleus, with the exception of *mre11*Δ mutant in the presence of HU, coherently with the lack of sensitivity of this mutant to HU observed in the plate assay showed above.

These observations strongly suggested that 9-1-1 and MRN complexes, as well as the claspin-like Mrc1, are required for the activation of Chk1 under different types of genotoxic stress.

### Mrc1, but neither 9-1-1 nor MRN, is required for the G2 arrest after the b-factor induction, as well as for full infection symptoms

**Fig 4:**
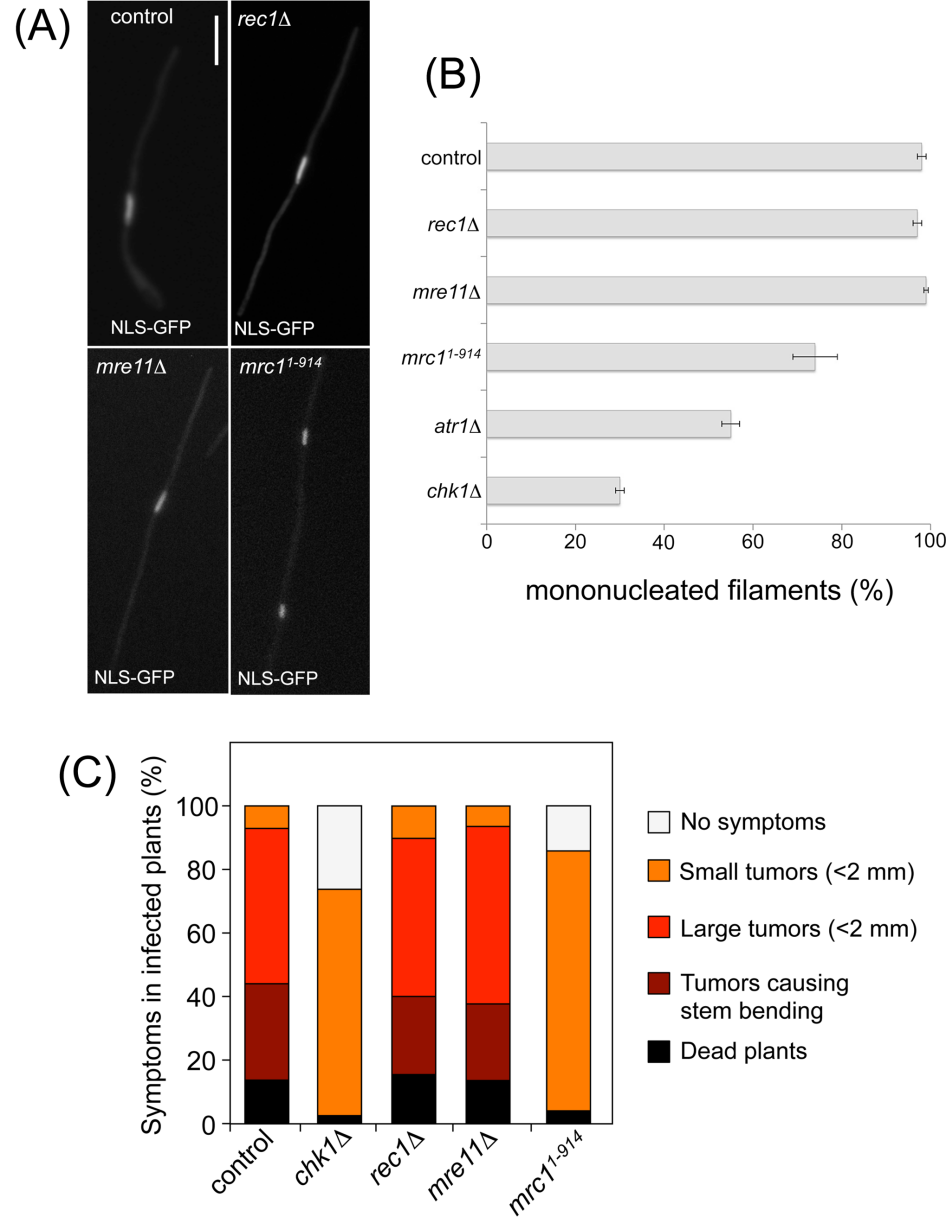
MRN and 9-1-1 complexes are dispensable for virulence and b-dependent cell cycle arrest. (A) Cell images of control (UMP112) and derived strains carrying the indicated mutations incubated for 8 h in inducing conditions (MMD). Strains carried an NLS-GFP fusion under control of the b-dependent *dik6* promoter to detect the nucleus. Bar: 20 μm. (B) AB33-derived strains carrying the *P*_*dik6*_:*NLS-GFP* transgene and carrying the indicated mutations were incubated in inducing conditions (MMD) for 8 hours. Filaments were sorted as carrying 1 or 2 and more nuclei. The graph shows the result from two independent experiments, counting more than 50 filaments each. Means and SDs are shown. (C) Disease symptoms caused by the indicated crosses were scored 14 days after infection of 7-day-old maize seedlings. Symptoms were grouped into color-coded categories depicted on the right side of graph. Two independent experiments were carried out and the average values are expressed as percentage of the total number of infected plants (n: > 50 plants).

Having demonstrated that *rec1*, *mre11* and *mrc1* genes were required for Chk1 activation upon induced genotoxic stress, we sought to address the question of whether they were also required for the cell cycle arrest that takes place upon *b*-induction. To test this possibility we introduced the *rec1*Δ, *mre11*Δ and *mrc1* ^*1-914*^ alleles into the UMP112 strain, which is derived from AB33 and carries under the control of the *dik6* promoter, a GFP fused to a nuclear localization signal. The expression of *dik6* promoter is dependent on an active b heterodimer [25], and therefore it allowed us the use of the nuclear fluorescence as a marker of the release of cell cycle arrest (counting the nuclear content of the filaments) as well as a surrogated marker of the ability of the different mutants to respond to the *b*-program. We have found no difference in the proportion of cells responding to b-factor in the different mutant backgrounds. Even at short times upon induction of *b* expression (4 h), we have observed that almost the whole cell population shows nuclear fluorescence, indicating that there might be no interferences with the *b*-induced transcriptional program. Strikingly, we have found that both Rec1 and Mre11 were dispensable for *b*-dependent cell cycle arrest: No differences between control (UMP112) filaments and filaments carrying the *rec1*Δ *and mre11*Δ alleles were found regarding nuclear content (Fig 4A and 4B). In contrast, in filaments carrying the *mrc1* ^*1-914*^ allele, it was possible to observe two and, less frequently, three nuclei, indicating that they are able to divide at least once, similarly as described for *chk1* and *atr1* mutants [15, 16].

Cells defective in Atr1 and Chk1 functions are affected in their pathogenic capability [15, 16]. Therefore we also investigated whether *rec1*Δ, *mre11*Δ and *mrc1* ^*1-914*^ mutants are able to infect maize. In *U*. *maydis*, virulence and sexual development are intricately interconnected. A prerequisite for generating the infectious stage is the mating of two compatible budding haploid cells to generate, after cell fusion, the infective dikaryotic filament. We constructed compatible haploid strains (i. e. *a1b1* and *a2b2* mating types) carrying the distinct mutant alleles. Mixtures of the respective mutant compatible strains, as well as wild-type controls, were used to infect seven-day-old maize seedlings by stem injection. The severity of disease symptoms was then scored 14 days after infection [24] (Fig 4C). We have found that the infection with strains carrying either *rec1*Δ or *mre11*Δ alleles were indistinguishable from those with wild-type cells, while infection with strains carrying the *mrc1* ^*1-914*^ allele was less efficient and never produced large tumors, as previously described for *chk1* and *atr1* mutants [15, 16].

Altogether, these results indicated that while the scaffold Claspin-like Mrc1 protein may be required for b-induced cell cycle arrest and virulence of *U*. *maydis*, the DDR upstream regulators Rec1 (9-1-1 complex) and Mre11 (MRN complex) seem dispensable for these functions.

## Discussion

During the infection of corn plants by *U*. *maydis*, the fungal cell cycle is arrested at G2 phase while it is growing on plant surface. This cell cycle arrest is required for the infective process, as mutants unable to stop the cell cycle progression are severely impaired in virulence [13, 15, 16]. Importantly, cell cycle arrest is dependent on the activation of the Atr1/Chk1 cascade, which would imply the presence or induction of some sort of DNA damage during this process. The aim of this work was to find any evidence of the DNA damage signal that would be feeding the ATR1/Chk1 cascade during the infective filament formation upon the b-factor expression in *U*. *maydis*. However, our results do not support the presence of the claimed DNA damage signal. We did not observe any signal of DNA damage in the form of RPA-GFP foci. Likewise, no obvious difficulties were found during S phase. Furthermore, the fact that neither 9-1-1 nor MRN, the two main sensors and transducers of DNA damage signals, were required for the *b*-induced cell cycle arrest as well as for virulence, also supports that no DNA damage signal would be feeding the Atr1/Chk1 cascade during the formation of the infective filament. It might also be plausible that induced and very localized DNA lesions could be recognized by alternative unknown sensors able to activate the checkpoint response. However, at this stage, we are much in favour of the hypothesis that Atr1/Chk1 activation during the development of the infective filament is independent of the presence of DNA damage as signaling cascade inducer.

Our analyses indicate that, although no upstream complexes such as MRN and 9-1-1 may be required, the adaptor protein Mrc1/Claspin seems necessary. Studies in yeast cells have shown that colocalization of Mec1 (Atr1) and Mrc1 is sufficient to induce the phosphorylation of the downstream kinase Rad53 *in vivo* [45]. More importantly, in this report, authors showed that this phosphorylation is independent of upstream signaling components. It could be possible that in *U*. *maydis*, upon activation of the genetic program responsible for the infective filament formation, Atr1 would interact with Mrc1 in a DNA-damage independent manner and thereby the complex would activate Chk1.

If no DNA damage signal is associated with the *b*-induced activation of the Atr1/Chk1 cascade, how might this activation take place? We envisioned several possibilities based on recent reports that indicate the possibility of activation of this pathway in a DNA-damage independent manner in other systems.

The first of these possibilities could be related with some altered chromatin conformation associated with the transcriptional program induced by the b-factor. Recent work proved that chromatin compaction was able to induce DDR in mammalian cells in the absence of any DNA damage [46]. So far, no studies about the chromatin state of *U*. *maydis* nuclei during the formation of the infective filament have been carried out, and therefore we cannot assure that such a chromatin compaction is taking place.

An alternative possibility is related to the stretch the nuclear envelope could be suffering during the formation of the infective filament. At this stage, the *U*. *maydis* cell experiments a strong induction of the polar growth, which is also dependent on the activation of the b-factor. During this elongation phase, the nuclei maintain a central position in the filament and for that it has to travel along the cytoplasm using microtubule-based motors, anchored to the nuclear pores [47]. Most likely, throughout this process the nuclear envelope is submitted to strong tensional forces and in fact nuclei inside the filament adopt a stretched appearance. In mammalian cells it has been reported that ATR activity at the nuclear envelope responds to mechanical stress. This ATR-mediated mechanical response occurs within the range of physiological forces, it is reversible, and it is independent of DNA damage signaling [48]. It could well be that in the case of *U*. *maydis*, the nuclear envelope deformation activated the Atr1/Chk1 cascade.

Although further work will be required to address these possibilities, our results reinforce the emerging idea that along evolution, the DDR cascade has been recruited to modulate developmental processes, most likely through its interaction with cell cycle machinery, even if no DNA damage signal exists.

## Supporting Information

S1 Fig (TIF)

S2 Fig (TIF)

## References

[1] Sherman MH, Bassing CH, Teitell MA. Regulation of cell differentiation by the DNA damage response. Trends Cell Biol. 2011;21(5):312–9. Epub 2011/03/01. 10.1016/j.tcb.2011.01.004 21354798 PMC3089693

[2] Bredemeyer AL, Helmink BA, Innes CL, Calderon B, McGinnis LM, Mahowald GK, et al DNA double-strand breaks activate a multi-functional genetic program in developing lymphocytes. Nature. 2008;456(7223):819–23. Epub 2008/10/14. 10.1038/nature07392 18849970 PMC2605662

[3] Egel R. Fission yeast mating-type switching: programmed damage and repair. DNA Repair (Amst). 2005;4(5):525–36. Epub 2005/04/07. 10.1016/j.dnarep.2004.11.004 . 15811625

[4] Haber JE. Mating-type genes and MAT switching in Saccharomyces cerevisiae. Genetics. 2012;191(1):33–64. Epub 2012/05/05. 10.1534/genetics.111.134577 22555442 PMC3338269

[5] Di Giovanni S, Knights CD, Rao M, Yakovlev A, Beers J, Catania J, et al The tumor suppressor protein p53 is required for neurite outgrowth and axon regeneration. Embo J. 2006;25(17):4084–96. Epub 2006/09/02. 10.1038/sj.emboj.7601292 16946709 PMC1560361

[6] Furuya K, Niki H. The DNA damage checkpoint regulates a transition between yeast and hyphal growth in Schizosaccharomyces japonicus. Mol Cell Biol. 2010;30(12):2909–17. Epub 2010/04/07. 10.1128/MCB.00049-10 20368354 PMC2876669

[7] Brauchle M, Baumer K, Gonczy P. Differential activation of the DNA replication checkpoint contributes to asynchrony of cell division in C. elegans embryos. Curr Biol. 2003;13(10):819–27. Epub 2003/05/16. . 12747829 10.1016/s0960-9822(03)00295-1

[8] Ullah Z, de Renty C, DePamphilis ML. Checkpoint kinase 1 prevents cell cycle exit linked to terminal cell differentiation. Mol Cell Biol. 2011;31(19):4129–43. Epub 2011/07/28. 10.1128/MCB.05723-11 21791608 PMC3187358

[9] Budirahardja Y, Gonczy P. Coupling the cell cycle to development. Development. 2009;136(17):2861–72. Epub 2009/08/12. 10.1242/dev.021931 . 19666818

[10] Perez-Martin J. Cell cycle and morphogenesis connections during the formation of the infective filament in Ustilago maydis In: Perez-Martin J, di Pietro A, editors. Morphogenesis and Pathogenicity in Fungi. Topics in Current Genetics. Berlin Heidelberg: Springer-Verlag; 2012 p. 97–114.

[11] Perez-Martin J, Castillo-Lluva S. Connections between polar growth and cell cycle arrest during the induction of the virulence program in the phytopathogenic fungus Ustilago maydis. Plant Signal Behav. 2008;3(7):480–1. Epub 2009/08/26. ; PubMed Central PMCID: PMC2634436. 19704492 10.4161/psb.3.7.5680 PMC2634436

[12] Perez-Martin J, de Sena-Tomas C. Dikaryotic cell cycle in the phytopathogenic fungus Ustilago maydis is controlled by the DNA damage response cascade. Plant Signal Behav. 2011;6(10):1574–7. Epub 2011/09/16. 10.4161/psb.6.10.17055 21918381 PMC3256387

[13] Castanheira S, Mielnichuk N, Perez-Martin J. Programmed cell cycle arrest is required for infection of corn plants by the fungus Ustilago maydis. Development. 2014 Epub 2014/11/21. 10.1242/dev.113415 . 25411209

[14] Perez-Martin J. DNA-damage response in the basidiomycete fungus Ustilago maydis relies in a sole Chk1-like kinase. DNA Repair (Amst). 2009;8(6):720–31. Epub 2009/03/10. doi: S1568-7864(09)00045-7 [pii] 10.1016/j.dnarep.2009.01.023 . 19269260

[15] Mielnichuk N, Sgarlata C, Perez-Martin J. A role for the DNA-damage checkpoint kinase Chk1 in the virulence program of the fungus Ustilago maydis. J Cell Sci. 2009;122(Pt 22):4130–40. Epub 2009/10/29. doi: jcs.052233 [pii] 10.1242/jcs.052233 . 19861497

[16] De Sena-Tomás C, Fernández-Álvarez A, Holloman WK, Pérez-Martín J. The DNA damage response signalling cascade regulates proliferation of the phytopathogenic fungus Ustilago maydis in planta. The Plant Cell. 2011;23:1654–65. 10.1105/tpc.110.082552 21478441 PMC3101559

[17] Feldbrugge M, Kamper J, Steinberg G, Kahmann R. Regulation of mating and pathogenic development in Ustilago maydis. Curr Opin Microbiol. 2004;7(6):666–72. Epub 2004/11/24. doi: S1369-5274(04)00130-4 [pii] 10.1016/j.mib.2004.10.006 . 15556041

[18] Banuett F, Herskowitz I. Different a alleles of Ustilago maydis are necessary for maintenance of filamentous growth but not for meiosis. Proc Natl Acad Sci U S A. 1989;86(15):5878–82. Epub 1989/08/01. . 16594058 10.1073/pnas.86.15.5878 PMC297734

[19] Holliday R. Ustilago maydis In: King RC, editor. Handbook of Genetics. New York: Plenum Press; 1974 p. 575–95.

[20] García-Muse T, Steinberg G, Pérez-Martín J. Pheromone-induced G2 arrest in the phytopathogenic fungus Ustilago maydis. Eukaryotic cell. 2003;2(3):494–500. 12796294 10.1128/EC.2.3.494-500.2003 PMC161457

[21] Tsukuda T, Bauchwitz R, Holloman WK. Isolation of the REC1 gene controlling recombination in Ustilago maydis. Gene. 1989;85(2):335–41. Epub 1989/12/28. . 2628171 10.1016/0378-1119(89)90426-5

[22] Brachmann A KJ, Julius C, Feldbrügge M. A reverse genetic approach for generating gene replacement mutants in Ustilago maydis. Molecular Genetics and Genomics. 2004;272(2):216–26. 15316769 10.1007/s00438-004-1047-z

[23] Becht P, Konig J, Feldbrugge M. The RNA-binding protein Rrm4 is essential for polarity in Ustilago maydis and shuttles along microtubules. J Cell Sci. 2006;119(Pt 23):4964–73. Epub 2006/11/16. 10.1242/jcs.03287 . 17105762

[24] Kamper J, Kahmann R, Bolker M, Ma LJ, Brefort T, Saville BJ, et al Insights from the genome of the biotrophic fungal plant pathogen Ustilago maydis. Nature. 2006;444(7115):97–101. Epub 2006/11/03. 10.1038/nature05248 . 17080091

[25] Brachmann A, Weinzierl G, Kamper J, Kahmann R. Identification of genes in the bW/bE regulatory cascade in Ustilago maydis. Mol Microbiol. 2001;42(4):1047–63. Epub 2001/12/12. doi: 2699 [pii]. . 11737646 10.1046/j.1365-2958.2001.02699.x

[26] Heimel K, Scherer M, Vranes M, Wahl R, Pothiratana C, Schuler D, et al The transcription factor Rbf1 is the master regulator for b-mating type controlled pathogenic development in Ustilago maydis . PLoS pathogens. 2010;6(8):e1001035 Epub 2010/08/12. 10.1371/journal.ppat.1001035 20700446 PMC2916880

[27] Heimel K, Scherer M., Vranes M, Wahl R, Pothiratana C, Schuler D, Vincon V, Finkernagel F, Flor-Parra I, Kämper J. The transcription factor Rbf1 is the master regulator for b-mating type controlled pathogenic development in Ustilago maydis. PLoS Pathogen 2010;6(8):e1001035. 20700446 10.1371/journal.ppat.1001035 PMC2916880

[28] Sgarlata C, Perez-Martin J. Inhibitory phosphorylation of a mitotic cyclin-dependent kinase regulates the morphogenesis, cell size and virulence of the smut fungus Ustilago maydis. J Cell Sci. 2005;118(Pt 16):3607–22. Epub 2005/07/28. 10.1242/jcs.02499 . 16046476

[29] Tarsounas M, Davies AA, West SC. RAD51 localization and activation following DNA damage. Philos Trans R Soc Lond B Biol Sci. 2004;359(1441):87–93. Epub 2004/04/07. 10.1098/rstb.2003.1368 15065660 PMC1693300

[30] Navadgi-Patil VMB, Burgers PM. A tale of two tails: Activation of DNA damage checkpoint kinase Mec1/ATR by the 9-1-1 clamp and by Dpb11/TopBP1. DNA Repair (Amst). 2009;8:996–1003. 19464966 10.1016/j.dnarep.2009.03.011 PMC2725207

[31] Raderschall E, Golub EI, Haaf T. Nuclear foci of mammalian recombination proteins are located at single-stranded DNA regions formed after DNA damage. Proceedings of the National Academy of Sciences of the United States of America. 1999;96(5):1921–6. Epub 1999/03/03. ; PubMed Central PMCID: PMC26712. 10051570 10.1073/pnas.96.5.1921 PMC26712

[32] Fan J, Pavletich NP. Structure and conformational change of a replication protein A heterotrimer bound to ssDNA. Genes Dev. 2012;26(20):2337–47. Epub 2012/10/17. 10.1101/gad.194787.112 23070815 PMC3475805

[33] Majka J, Niedziela-Majka A, Burgers PMJ. The Checkpoint Clamp Activates Mec1 Kinase during Initiation of the DNA Damage Checkpoint. Mol Cell. 2006;24:891–901. 17189191 10.1016/j.molcel.2006.11.027 PMC1850967

[34] Gobbini E, Cesena D, Galbiati A, Lockhart A, Longhese MP. Interplays between ATM/Tel1 and ATR/Mec1 in sensing and signaling DNA double-strand breaks. DNA Repair (Amst). 2013;12(10):791–9. Epub 2013/08/21. 10.1016/j.dnarep.2013.07.009 . 23953933

[35] Alcasabas AA, Osborn AJ, Bachant J, Hu F, Werler PJ, Bousset K, et al Mrc1 transduces signals of DNA replication stress to activate Rad53. Nat Cell Biol. 2001;3(11):958–65. Epub 2001/11/21. 10.1038/ncb1101-958 . 11715016

[36] Schwartz MF, Duong JK, Sun Z, Morrow JS, Pradhan D, Stern DF. Rad9 phosphorylation sites couple Rad53 to the Saccharomyces cerevisiae DNA damage checkpoint. Mol Cell. 2002;9(5):1055–65. Epub 2002/06/07. . 12049741 10.1016/s1097-2765(02)00532-4

[37] Tanaka K, Russell P. Mrc1 channels the DNA replication arrest signal to checkpoint kinase Cds1. Nat Cell Biol. 2001;3(11):966–72. Epub 2001/11/21. 10.1038/ncb1101-966 . 11715017

[38] de Sena-Tomas C, Yu EY, Calzada A, Holloman W, Lue N, Perez-Martin J. Fungal Ku prevents permanent cell cycle arrest by suppressing DNA damage signaling at telomeres. Nucleic Acid Res. 2015 10.1093/nar/gkv082 PMC4344518 25653166

[39] Onel K, Koff A, Bennett RL, Unrau P, Holloman WK. The REC1 gene of Ustilago maydis, which encodes a 3'—>5' exonuclease, couples DNA repair and completion of DNA synthesis to a mitotic checkpoint. Genetics. 1996;143(1):165–74. Epub 1996/05/01. ; PubMed Central PMCID: PMC1207251. 8722772 10.1093/genetics/143.1.165 PMC1207251

[40] Garcia V, Furuya K, Carr AM. Identification and functional analysis of TopBP1 and its homologs. DNA Repair (Amst). 2005;4(11):1227–39. Epub 2005/05/18. 10.1016/j.dnarep.2005.04.001 . 15897014

[41] Tanaka K. Multiple functions of the S-phase checkpoint mediator. Biosci Biotechnol Biochem. 2010;74(12):2367–73. Epub 2010/12/15. 10.1271/bbb.100583 . 21150122

[42] Araki H, Leem SH, Phongdara A, Sugino A. Dpb11, which interacts with DNA polymerase II(epsilon) in Saccharomyces cerevisiae, has a dual role in S-phase progression and at a cell cycle checkpoint. Proceedings of the National Academy of Sciences of the United States of America. 1995;92(25):11791–5. Epub 1995/12/05. ; PubMed Central PMCID: PMC40488. 8524850 10.1073/pnas.92.25.11791 PMC40488

[43] Osborn AJ, Elledge SJ. Mrc1 is a replication fork component whose phosphorylation in response to DNA replication stress activates Rad53. Genes Dev. 2003;17(14):1755–67. Epub 2003/07/17. 10.1101/gad.1098303 12865299 PMC196183

[44] Ciccia A, Elledge SJ. The DNA damage response: making it safe to play with knives. Mol Cell. 2010;40(2):179–204. Epub 2010/10/23. 10.1016/j.molcel.2010.09.019 20965415 PMC2988877

[45] Berens TJ, Toczyski DP. Colocalization of Mec1 and Mrc1 is sufficient for Rad53 phosphorylation in vivo. Mol Biol Cell. 2012;23(6):1058–67. Epub 2012/02/03. 10.1091/mbc.E11-10-0852 22298423 PMC3302733

[46] Burgess RC, Burman B, Kruhlak MJ, Misteli T. Activation of DNA damage response signaling by condensed chromatin. Cell Rep. 2014;9(5):1703–17. Epub 2014/12/04. 10.1016/j.celrep.2014.10.060 25464843 PMC4267891

[47] Steinberg G, Schuster M, Theisen U, Kilaru S, Forge A, Martin-Urdiroz M. Motor-driven motility of fungal nuclear pores organizes chromosomes and fosters nucleocytoplasmic transport. J Cell Biol. 2012;198(3):343–55. Epub 2012/08/02. 10.1083/jcb.201201087 22851316 PMC3413351

[48] Kumar A, Mazzanti M, Mistrik M, Kosar M, Beznoussenko GV, Mironov AA, et al ATR mediates a checkpoint at the nuclear envelope in response to mechanical stress. Cell. 2014;158(3):633–46. Epub 2014/08/02. 10.1016/j.cell.2014.05.046 25083873 PMC4121522

