# Supplementary figures and images for: MRN- and 9-1-1-Independent Activation of the ATR-Chk1 Pathway during the Induction of the Virulence Program in the Phytopathogen Ustilago maydis
Source: PLoS One. 2015 Sep 14;10(9):e0137192. doi: 10.1371/journal.pone.0137192 (PMC4573213; doi:10.1371/journal.pone.0137192)

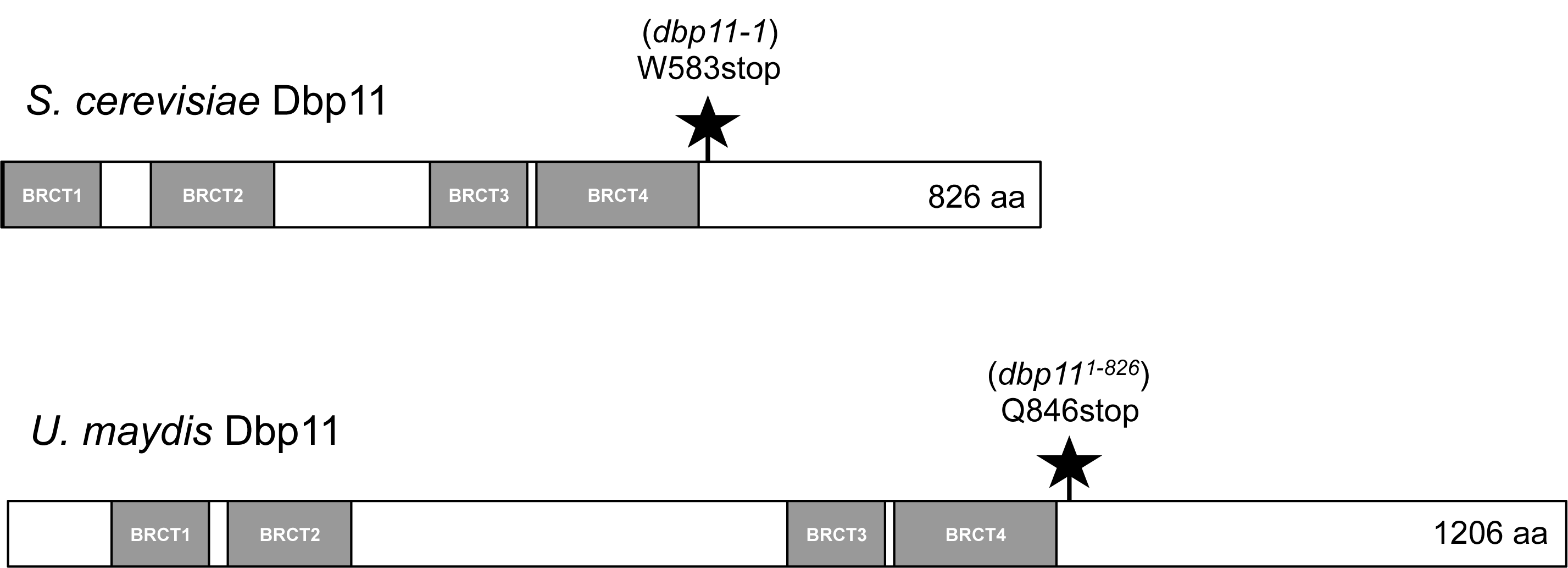

Supplement: S1 Fig — (TIF) [file pone.0137192.s001.tif]

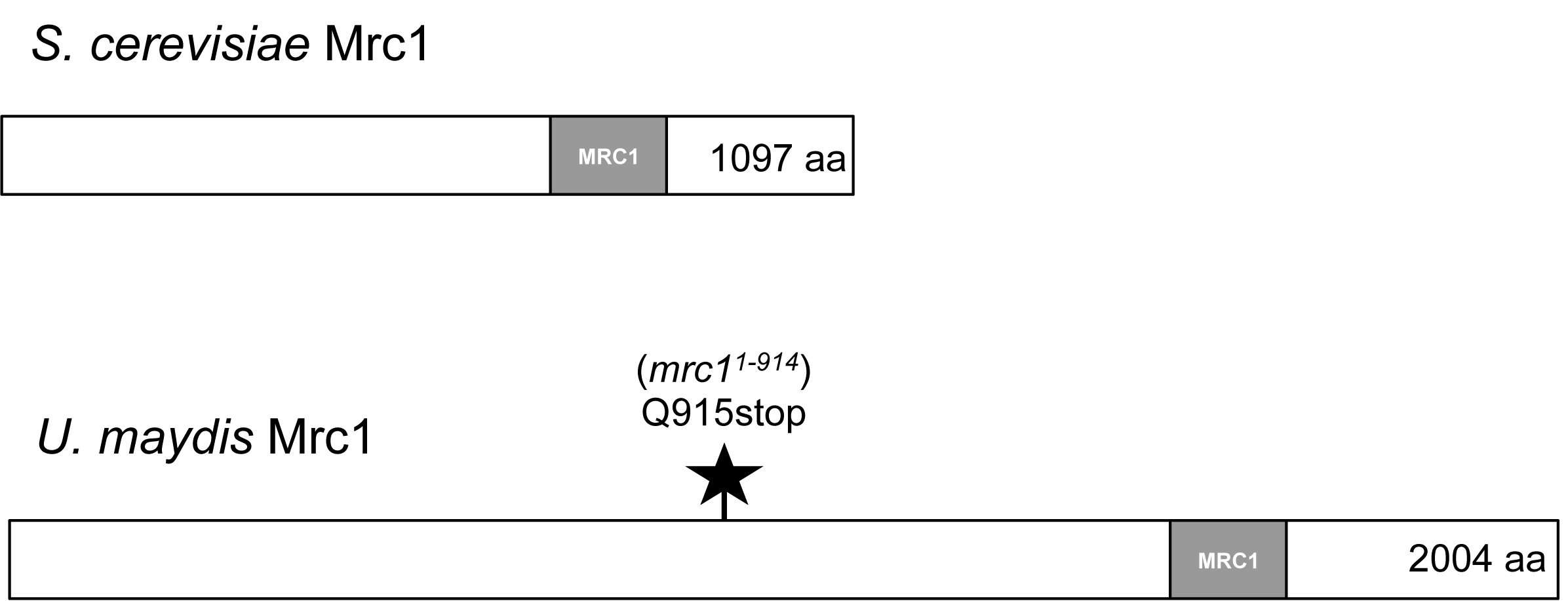

Supplement: S2 Fig — (TIF) [file pone.0137192.s002.tif]
